# First Proof-of-Principle of Inorganic Lead Halide Perovskites Deposition by Magnetron-Sputtering

**DOI:** 10.3390/nano10010060

**Published:** 2019-12-26

**Authors:** Claudia Borri, Nicola Calisi, Emanuele Galvanetto, Naomi Falsini, Francesco Biccari, Anna Vinattieri, Giuseppe Cucinotta, Stefano Caporali

**Affiliations:** 1 DIEF—Industrial Engineering Department, University of Florence, Via S. Marta 3, 50139 Florence, Italy; borricla@gmail.com (C.B.); nicola.calisi@unifi.it (N.C.); emanuele.galvanetto@unifi.it (E.G.); 2 INSTM—Interuniversity National Consortium for Material Science and Technology, Via Giusti 9, 50121 Florence, Italy; naomi.falsini@unifi.it (N.F.); anna.vinattieri@unifi.it (A.V.); 3 Department of Physics and Astronomy, University of Florence, via G. Sansone 1, 50019 Sesto Fiorentino (FI), Italy; francesco.biccari@unifi.it; 4 LENS—European Laboratory for Non-Linear Spectroscopy, via Nello Carrara 1, I-50019 Sesto Fiorentino (FI), Italy; 5 INFN—National Institute for Nuclear Physics, via G. Sansone 1, 50019 Sesto Fiorentino (FI), Italy; 6 Chemistry Department “U. Schiff”, University of Florence, via della Lastruccia 3, 50019 Sesto Fiorentino (FI), Italy; giuseppe.cucinotta@unifi.it

**Keywords:** perovskite, thin-film, magnetron-sputtering, caesium lead halides

## Abstract

The present work reports the application of RF-magnetron sputtering technique to realize CsPbBr${}_{3}$ 70 nm thick films on glass substrate by means of a one-step procedure. The obtained films show highly uniform surface morphology and homogeneous thickness as evidenced by AFM and SEM investigations. XRD measurements demonstrate the presence of two phases: a dominant orthorhombic CsPbBr${}_{3}$ and a subordinate CsPb${}_{2}$Br${}_{5}$. Finally, XPS data reveals surface bromine depletion respect to the stoichiometrical CsPbBr${}_{3}$ composition, nevertheless photoluminescence spectroscopy results confirm the formation of a highly luminescent film. These preliminary results demonstrate that our approach could be of great relevance for easy fabrication of large area perovskite thin films. Future developments, based on this approach, may include the realization of multijunction solar cells and multicolor light emitting devices.

## 1. Introduction

In the last decade, halide perovskites have attracted the attention of the scientific community thanks to their excellent optoelectronic properties [1,2,3,4] which make them suitable for applications ranging from light emitting diodes to nanotechnologies [5]. Among the materials proposed for optoelectronic devices, halide perovskite materials present several advantages such as the relatively low-cost, the possibility to tune the band gap varying the composition, and the different kind of nanostructure obtainable. For such reasons, halide perovskites have been recently proposed as novel materials for solar cells, sensors and LEDs [3,6,7,8,9,10].

Historically, the first proposed halide perovskites were constituted by organic and inorganic compounds based on methylammonium lead trihalide (MAPbX${}_{3}$, with X = Cl, Br, I). However, these materials have a major drawback in their high chemical instability that leads to a significant degradation of the materials in short times, especially after exposure to humidity, UV light and moderately high temperature [11]. This instability makes them unsuitable for application where a long lifetime is crucial. To overcome this issue, fully inorganic perovskites, which present comparable optoelectronics properties to the hybrid ones, were realized by the substitution of the organic cation by an inorganic one [12]. Solution-based chemical synthesis represents the most common route used for deposition of thin and thick layers and, among them, spin-coating and dipping techniques are the most widely used [5]. However, these approaches are valuable for assembling small laboratory samples but result less suitable for the production of devices of several square centimeters, or larger, as required for industrial applications. Blade coating, spray coating and vapor depositions methods are more suitable for this purpose and the progress toward the deposition of large-area perovskites have been recently reviewed [13,14]. Nevertheless, it is an open challenge the search for a techniques that allows the deposition of several layers of different material (perovskites, scaffold layers, hole and electron transport layers, metal electrodes) with controlled characteristics (morphology, thickness, roughness, uniformity) as it’s required for device fabrication.

In this context radio frequency (RF) magnetron sputtering constitutes a powerful technique for the deposition of both, conductive (metals) and non-conductive (ceramic) materials [15,16] thin films, offering the possibility to grow consecutive layers of different materials with a nanometric control of the thickness and a reduced surface roughness. Other advantages are the homogeneity of the obtained films, the limited contamination from unwanted elements and, conversely, the ease of doping. In addition the growth can be performed at room temperature avoiding, or at least limiting, the material stress: this latter is a consequence of the large mismatch between thermal expansion coefficient (CTE) of the perovskites (3.8 × 10${}^{-5}$ K${}^{-1}$ for CsPbBr${}_{3}$ at room temperature [17]) and the substrate (typically CTE for glass substrates < 6 × 10${}^{-6}$ K${}^{-1}$) and could be responsible of the layers cracking and degradation [18,19,20]. Despite its advantages, it has received a very limited attention; to our knowledge only two studies report on the depositions of hybrid halide perovskite layers (i.e., CH${}_{3}$NH${}_{3}$PbI${}_{3}$) [21,22], while there are no attempts to deposit fully inorganic lead perovskites.

In this study, we report the successful direct deposition of thin films of CsPbBr${}_{3}$ by one step magneto-sputtering. Even if there is evidence of the presence of two distinct phases with different stoichiometrical ratio between Cs, Pb and Br, the structural, optical, and electrical properties of the prepared samples are comparable, and in some case result more performing, then the usual solution-based grown inorganic perovskites.

## 2. Materials and Methods

**Figure 1:**
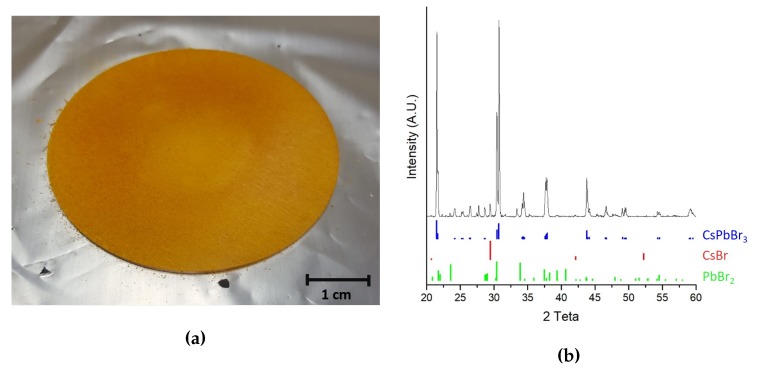
(**a**) Picture of the obtained perovskite target (**b**) XRD spectra of the obtained perovskite powder.

CsPbBr${}_{3}$ powder was obtained through a mechanochemical procedure described in literature [23]. It consists by grinding the two precursor salts (CsBr and PbBr${}_{2}$ purchased from Merck KGaA, Darmstadt, Germany) in equal molar ratio in a mixer mill (Retsch model MM400, Haan, Germany). The success of the synthesis was qualitatively witnessed by the color change of the powder from white to yellow (see Figure 1a) and, more quantitatively, by powder XRD pattern which also confirmed the absence of unreacted precursors (see Figure 1b).

The sputtering target (5 cm diameter disk) was realized by pressing the perovskite powder by means of a pneumatic press (11.5 MPa working pressure) for 24 h at room temperature. The so obtained target is depicted in Figure 1a.

The magnetron sputtering equipment is constituted by a Korvus HEX system, (Korvus Technology Ltd., Newington, UK) coupled with an RF source working at 13.56 MHz. The deposition was performed at room temperature with an RF power of 20 W and argon gas flow of 20 atm cm${}^{3}$ min${}^{-1}$. In these conditions, the dynamic working pressure was 2 × 10${}^{-6}$ atm and the deposition rate resulted to be 7 × 10${}^{-2}$ nm s${}^{-1}$. We monitored the film thickness by using a quartz crystal nanobalance until it reached the desired thickness of 70 nm after about 17 min. During this time, the sample holder was kept rotating to assure thickness homogeneity. At the end of the sputtering procedure, the obtained film resulted transparent, uniform and slightly orange colored, with a green fluorescence under a 254 nm UV lamp.

Scanning electron microscopy (SEM, Gaia3 FESEM, Tescan Orsay Holding, Brno - Kohoutovice, Czech Republic) was performed to assess the morphology, the homogeneity and the thickness of the film. To obtain an optimal cross section of the film, an in-situ platinum deposition was performed before the focused ion beam cutting. Unfortunately, due to the small film thickness of the film chemical composition could not be measured by energy dispersive spectroscopy (EDS). Surface roughness and morphology was investigated by atomic force microscopy (AFM). Morphological analysis have been carried out with a NT-MDT Solver P47pro Scanning Probe Microscope (NT-MDT, Zelenograd, Moscow, Russia). AFM images were recorded in tapping mode in air using a HQ:NSC36B/Al BS N-type silicon tip with a resonance frequency of 130 KHz and we used the Gwyddion, open source software, version 2.54 to perform the statistical analysis on the AFM obtained data.

Due to the small thickness, the chemical composition of the film could not be determined by EDS, therefore, chemical investigation was performed by X-ray photoelectron spectroscopy (XPS). We used an XPS instrument constituted by an X-ray source (VSW Scientific Instrument Limited model TA10, Al K${}_{\alpha }$ radiation, 1486.6 eV) and a hemispherical analyzer model HA100 (VSW Scientific Instrument Limited, Manchester, UK) with a 16 channels detector. The XPS peaks were fitted by CasaXPS software after Shirley’s type background substraction [24]. The binding energies (BEs) values were calibrated using as internal reference the 1s transition of adventitious carbon fixed at 284.8 eV [25]. Peaks were fitted by Gaussian-Lorentzian components, imposing for each doublet the distance between the two peaks and the area ratio.

The structural investigation was achieved by grazing angle XRD carried out with a Bruker model D8 Advance diffractometer. Diffuse reflectance spectroscopy (DRS, Agilent Cary 300 spectrophotometer, equipped with a Labsphere PELA-1050 integration sphere) measurements assessed the direct band-gap value by McLean analysis at the absorption edge [26].

The photoluminescence (PL) measurements were performed in a quasi-backscattering geometry (illuminated area about 100 μm). The samples were kept in a closed cycle cryostat, allowing to span a temperature range from 10 to 300 K. Continuous wave (CW) measurements were performed exciting the samples by a laser diode operating at 405 nm with an excitation intensity of about 10 W cm${}^{-2}$, while a frequency-doubled mode-locked ps Ti:Sapphire laser, operating at 81.3 MHz repetition rate with 1.2 ps pulses, was used for time-resolved (TR) experiments. The PL signal was spectrally dispersed by a monochromator (Acton SpectraPro HRS-300, grating 300 gr/mm blazed at 500 nm) providing a spectral resolution of 1 meV and detected by by a streak camera Hamamatsu model C5680 (Hamamatsu Photonics, Hamamatsu City, Shizuoka, Japan) (time resolution 5 ps).

## 3. Results

### 3.1. Morphological Characterization

**Figure 2:**
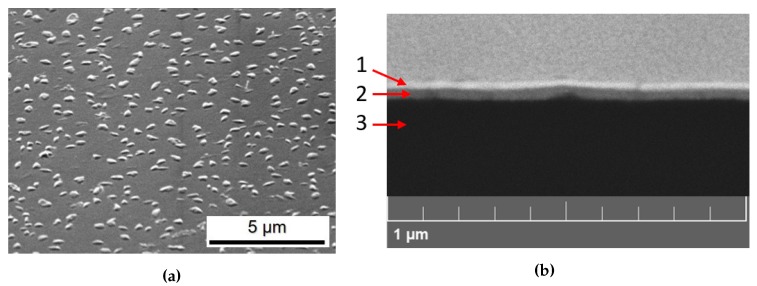
SEM images of a deposited CsPbBr_3_ sample on glass. (**a**) Secondary Electrons micrograph. (**b**) Backscattered electrons micrograph of a sample cross section. Three layers are visible: the Pt coating (1), the 70 nm sputtered film (2), the glass substrate (3).

The SEM micrograph displayed in Figure 2a depicts a compact and well-distributed film on which numerous nanocrystals (about 100 nm size) are uniformly distributed all over the sample.

The Back Scattered Electron (BSE) image of the cross section of the sample is shown in Figure 2b. The CsPbBr${}_{3}$ film appears to be very compact and homogeneous, about 70 nm thick, confirming the gravimetrical value given by the nanobalance.

**Figure 3:**
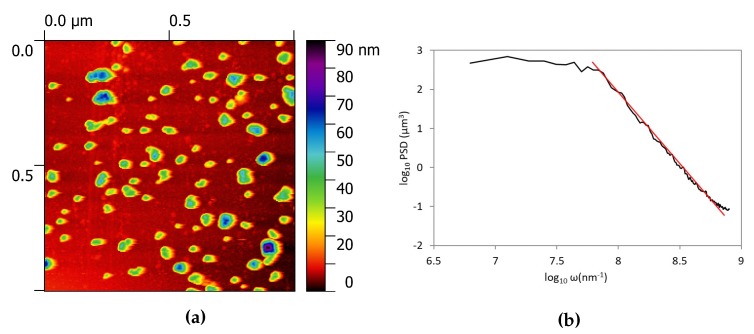
Surface data on a 1 × 1 μm zone. (**a**) is the AFM image and (**b**) is the power spectral density function evaluated in the same area.

Figure 3a reports the morphology of the sample obtained in a 1 × 1 μm area by AFM measurement. It is possible to observe the presence of small particles with height of about 40–50 nm. To be sure to have a representative sample of the overall morphology, we recorded the surface topography in 10 different spots and evaluated the roughness as a mean value of these measurements. The overall roughness, evaluated as the arithmetic average of the absolute values of the profile height deviations from the mean line of the deposit is 6.1 nm and the root means square 9.8 nm; these data asses the small roughness of the obtained deposits.

Historically, two approaches have been used to describe the characteristics of rough surfaces in order to understand their properties and model their behaviour. The 2D extension of the random process theory, previously applied to random noise signals, was proposed by Longuett-Higgins [27] and then extended to 3D to solve the contact problem by Nayak [28]. Meanwhile, the characteristics of self-affinity of natural rough surfaces emerged [29] and were object of new research. Hence, to better characterize the morphology of the film surface, we evaluated the power spectral density function (PSD) as the fast Fourier transform of the height fields [30]. It is known that, for fractal surfaces the PSD has a power law dependency on the frequency [31] and that the fractal dimension can be evaluated as the slope β of a least-square regression line fit to the data points in log-log plot of power spectrum as Df = 7/2 + β/2 [32,33,34]. In this case the fractal dimension was D = 2.2, that corresponds to a smooth surface. From the PSD plot depicted in Figure 3b, we can also identify the presence of a cut-off at lower frequency, usually this kind of feature is characteristic of a grinded or polished surface, on which the asperities larger than the grinding particle dimension have been eliminated. In our case it can be related to the maximum dimension of the particles present on the surface. The cut-off of the PSD is located at a frequency value of log${}_{10}$ω = 7.65 nm${}^{-1}$ that corresponds to a length scale of 23 nm, in agreement with the topography of the sample.

**Figure 4:**
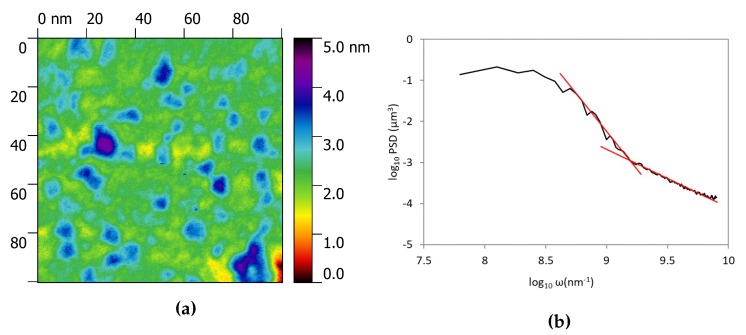
Surface data on a 100 × 100 nm zone. (**a**) is the AFM image and (**b**) is the power spectral density function evaluated in the same area.

**Figure 5:**
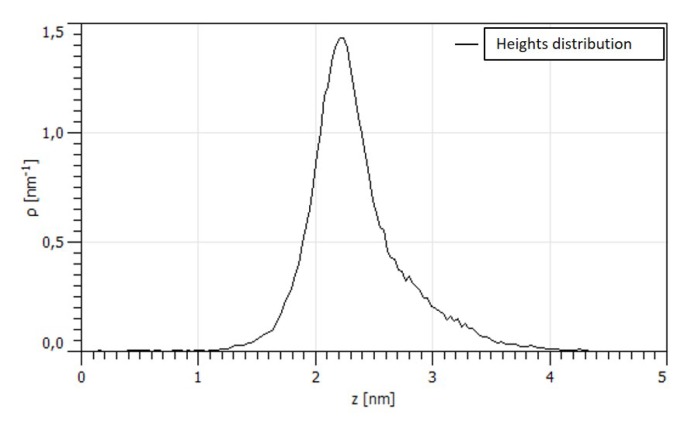
Distribution of asperity heights.

We also focused our attention on the flat smaller zones of the surface in between the larger crystals shown in Figure 3a to analyze the surface with a higher resolution. As an example one of the topography is reported in Figure 4a. The 100 × 100 nm zone shows the presence of much smaller asperities that were not evident in larger topography. Figure 4b shows a variation in the slope of the PSD evaluated in this zone. Higher slope in the high frequency range usually is associated with a texturing of the surface at the nano-scale level as previously reported [35]. This so called bifractality suggests two different level of organization in the surface and the shift between the two regimes is located in correspondence of 1 nm lenghtscale. The distribution of the asperity heights in Figure 5 shows that the medium height of the particles is 2 nm. We can suppose that the two phases present in the deposit have two different particle sizes and lenghtscale, however, it is difficult to prove this hypothesis since the small thickness of the film does not allow a EDS point analysis on the particles.

### 3.2. Chemical and Structural Characterization

The chemical analysis and phase identification of the deposited thin film were performed by XPS and XRD. The first one allows the evaluation of the chemical composition of the sample and thanks to its surface sensitivity (in such conditions the technique probing depth is about 4 nm) it is particularly suited for thin film analysis. The second one provides information concerning the lattice structure of the film and, by comparing results with tabulated data, the nature of the mineralogical species was attributed.

**Figure 6:**
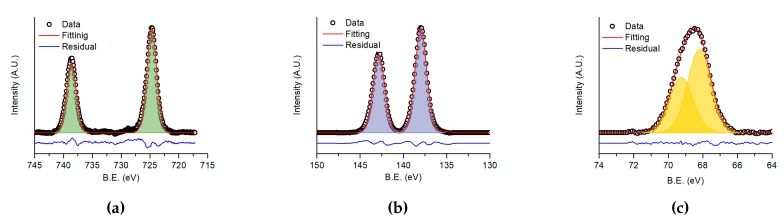
XPS spectra of (**a**) 3d transition of caesium, (**b**) 4f transition of lead and (**c**) 3d transition of bromine. The dots are the experimental data, the red line is the fitting curve and the blue line is the residual.

In Figure 6 the high-resolution XPS spectra of caesium 3d transition (from 745 eV to 720 eV, Figure 6a), lead 4f transition (from 150 eV to 130 eV, Figure 6b) and bromine 3d transition (from 74 eV to 64 eV, Figure 6c) are reported. The results of the fitting are listed in Table 1, where the calculated and theoretical atomic percentage contents of each element are reported.

The measured percentages were calculated from the relative XPS peaks after applying tabulated atomic sensitivity factors [36]. Experimental results were slightly different form the expected stoichiometrical values. In particular, an excess of caesium and a lack of bromine was observed in the deposed film. Reasonably, the elements have different sputtering rates as previously reported by Bonomi et al. for hybrid perovskites [22]. However, it is also known that halogen vacancies are the most abundant defect in CsPbX${}_{3}$ materials (see [37] and references therein). The presence of such defects, mostly related to the surface, is considered responsible of the changes in the PL intensity when samples are exposed to air and different gases [38]. To determine if bromine vacancies were located only in the surface region of the film, we carried out depth profiling XPS analysis. The same percentages of the elements with the respect to the surface was found throughout all the thickness. Since the starting materials returned the correct stoichiometrical composition, different sputtering rates should be responsible for the change in composition. Anyhow, the binding energies observed for each element (Table 1) are very similar to those observed in previous reported XPS analysis for CsPbBr${}_{3}$.

**Figure 7:**
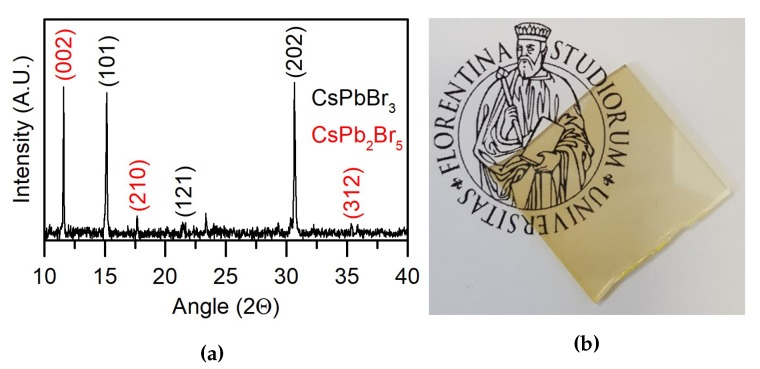
(**a**) XRD spectrum of the thin film. In black are evidenced the peaks of the CsPbBr_3_ phase and in red the peaks of the CsPbBr_5_ inclusions (**b**) Picture of the obtained transparent thin film.

A detailed information relative to the mineralogical nature of the deposited film, a picture of which is displayed in Figure 7b, was obtained by XRD. The spectrum (Figure 7a) displays peaks attributable to CsPbBr${}_{3}$ perovskite. However, their relative intensities do not fulfill the relative intensities achievable from a randomly oriented crystalline compound, indicating, on the whole, high texturing of the film. Peaks attributable to the phase CsPb${}_{2}$Br${}_{5}$ are also present (labeled in red in Figure 7a). This phase reasonably accounts for the bromine deficiency observed by XPS measurements. Further investigations will be carried out in order to assess the optimal conditions to avoid the formation of these spurious phases, for example varying the amount of CsBr in the sputtering target.

### 3.3. Optical Characterization

**Figure 8:**
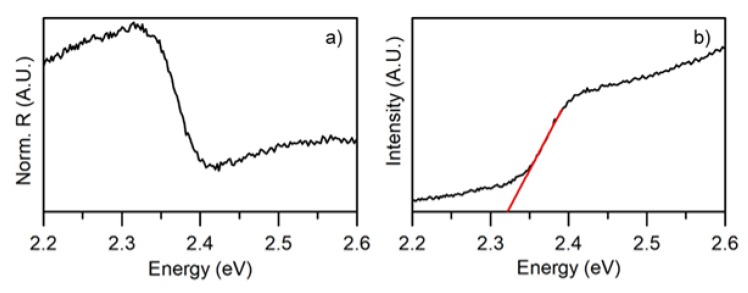
(**a**) Diffuse reflectance spectrum of the magnetron sputtered thin film on glass and (**b**) Band gap of the thin film calculated from absorbance data using the Tauc relation.

Figure 8 shows the DRS spectrum obtained on the sputtered film. The band-gap value can be estimated by fitting the McLean analysis at the absorption edge [26] i.e., by the extrapolation of the linear trend in the Tauc plot [39,40]. The direct band-gap resulted 2.31 eV, slightly smaller than the bulk CsPbBr${}_{3}$. In accordance to previous reports [41,42,43] the band-gap decrease could be related to the small thickness and crystallite size of the film.

**Figure 9:**
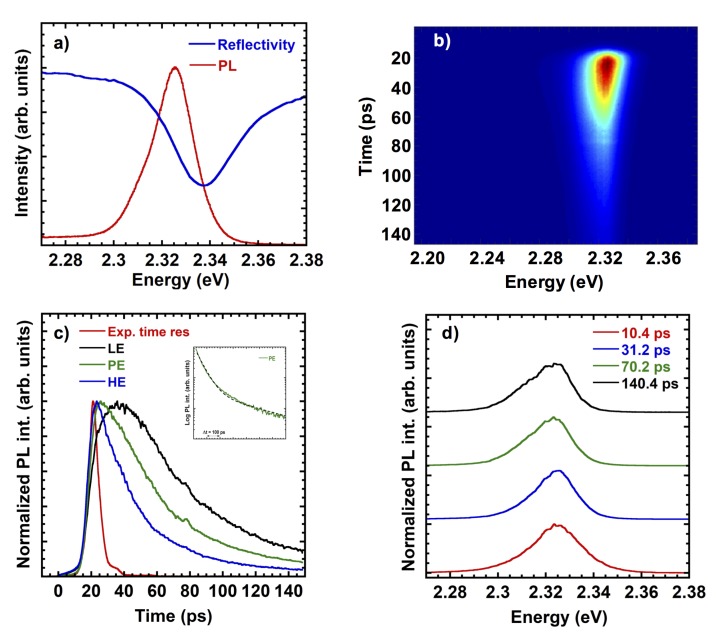
Photoluminescence spectra at 10 K. (**a**) CW PL and reflectivity spectrum. (**b**) Streak camera image of the PL. (**c**) Normalized PL decays at three different energies extracted from (**b**): HE-high energy side at 2.34 eV, PE-Peak energy at 2.325 eV, LE-low energy side at 2.31 eV. The red curve in (**c**) is the experimental time response. In the inset a fit of the PL decay (dashed line) at 2.325 eV is reported. (**d**) Time-resolved PL spectra extracted from (**b**).

PL experiments were performed from 10 K to 300 K: here we show in Figure 9 results at low temperature to assess the overall quality of the material. In fact our results indicate an inhomogeneous broadening comparable to results reported for spin-coated samples (<20 meV) and a small Stokes shift of the PL respect to the reflectivity spectrum, with a marked excitonic resonance. In Figure 9c the slow rise of the low energy emission accounts for the exciton localization, which is a process occurring in a longer time scale respect to the exciton formation/radiative recombination, as commonly found in literature when high quality semiconductor samples are investigated. Such localisation effect shows up in Figure 9d as an increase of the low energy contribution at the PL at longer delay. The low temperature PL spectrum and the reflectivity spectrum well agree with literature data on CsPbBr${}_{3}$ thin films [44]. The PL time evolution cannot be described by a single exponential, in agreement with literature data for perovskite. A fit of the PL decay at 2.325 eV is shown in the inset of Figure 9c: the dashed line is a two-exponential fit with 50 ± 10 ps and 400 ± 50 ps. The initial decay turns out to be slightly faster respect to values reported for pure CsPbBr${}_{3}$ spin-coated samples [44,45,46], therefore such result indicates that the presence of small amount of the CsPb${}_{2}$Br${}_{5}$ phase does not affect the PL emission. A slight localization is present with a shift of the PL peak energy less than 5 meV, while bound states appear on the low energy side, as the time delay increases. The overall PL behavior turns out to be comparable to state of the art spin coated thin films.

## 4. Conclusions

Here we demonstrate, for the first time in the literature, the successful deposition of thin (70 nm) CsPbBr${}_{3}$ films by RF-magnetron sputtering technique starting from a single perovskite target. The obtained film is uniform and transparent, highly textured and mainly constituted by CsPbBr${}_{3}$. The optical properties of the film are similar to bulk materials. Overall, in this paper we propose a new route to the fabrication of fully inorganic perovskite thin films, which could be applied as a new fabrication method for large size devices and multi-layer heterostructures, opening new and stimulating scenarios in the current research on perovskite-related functional materials. Future developments will include the optimization of the deposition parameters to better control the stoichiometry and the direct realization of perovskite solar cells by multi-layered magnetron-sputtering deposition.

## Figures and Tables

**Table 1:** Binding energies and measured peaks area for Cs, Pb and Br as evaluated from XPS data. The measured atomic percentage refers to the absolute elemental content as detected by the technique.

Element	B.E. (eV)	Area	Measured Atomic Percentage	Expected Atomic Percentage
Cs	724.7	3746	35 ± 3	20
Pb	138.0	10230	20 ± 2	20
Br	68.2	5661	45 ± 4	60

## Abbreviations

The following abbreviations are used in this manuscript:

BE	Binding energy
BSE	Backscattered
CTE	Thermal expansion coefficient
CW	Continuous wave
DRS	Direct reflectance spectroscopy
EDS	Energy dispersive spectroscopy
MA	Methyl ammonium
PSD	Power spectral density
RF	Radio frequency
XPS	X-ray photoelectron spectroscopy
XRD	X-ray diffraction
